# Comparison of Familial Clustering of Anogenital and Skin Cancers Between In Situ and Invasive Types

**DOI:** 10.1038/s41598-019-51651-6

**Published:** 2019-11-06

**Authors:** Luyao Zhang, Otto Hemminki, Guoqiao Zheng, Asta Försti, Kristina Sundquist, Jan Sundquist, Kari Hemminki

**Affiliations:** 1 Division of Molecular Genetic Epidemiology, German Cancer Research Center (DKFZ), Im Neuenheimer Feld 580, D-69120 Heidelberg, Germany; 2 Faculty of Medicine, University of Heidelberg, Heidelberg, Germany; 3 Department of Urology, Helsinki University Hospital, Helsinki, Finland; 4 Cancer Gene Therapy Group, Faculty of Medicine, University of Helsinki, Helsinki, Finland; 5 Center for Primary Health Care Research, Lund University, 205 02 Malmö, Sweden; 6 Department of Family Medicine and Community Health, Department of Population Health Science and Policy, Icahn School of Medicine at Mount Sinai, New York, USA; 7 Center for Community-based Healthcare Research and Education (CoHRE), Department of Functional Pathology, School of Medicine, Shimane University, Matsue, Japan

**Keywords:** Oncology, Risk factors

## Abstract

Literature on familial risk of carcinomas *in situ* (CISs) is limited because many cancer registries do not collect information on CIS. In Sweden CISs are collected, and we used these data to analyze familial relative risks (RRs) for concordant (CIS-CIS) types of anogenital (cervical, other female and male genital and anal) and skin squamous cell CIS; additionally RRs were assessed between CIS types and between CIS and invasive forms. RRs were calculated for the offspring generations when family members were diagnosed CIS. Case numbers for CIS ranged from 330 in anal to 177,285 in cervical CIS. Significant concordant CIS-CIS RRs were 2.74 for female genital, 1.77 for cervical and 2.29 for SCC skin CISs. The CIS forms associated also with each other, except for cervical and skin CIS types. RRs for concordant CIS-invasive cancer associations were lower than CIS-CIS associations. Cervical CIS associated with non-Hodgkin CIS which may suggest immune dysfunction as a contributing factors. The results for anogenital CIS types suggest that life style related human papilloma virus infections contributed to the observed familial associations. Lower risks for CIS-invasive cancer than CIS-CIS suggest that CIS and invasive cancers share only partially risk factors that underlie familial clustering.

## Introduction

Dysplasia is the earliest form of precancerous lesion and high-grade dysplasia is often synonymously referred to as carcinoma *in situ* (*in situ* cancer, CIS)^1^. CIS is a local accumulation of dysplastic cells. While CIS has not spread through its membranous borders, such as basement membrane, it may have the potential to do so and transform into cancer^1,2^. Because of cervical cancer screening programs, cervical CISs are very common and in many countries far outnumber invasive cervical cancers. Similarly, CIS for skin squamous cell (SCC) may outnumber the invasive SCCs in the skin^3,4^. Literature on CISs is limited because many cancer registries do not collect information on them. In Sweden, notifications of CISs to the cancer registry are mandated similar to invasive cancer, and these are included in the Swedish Family-Cancer Database^5^.

Considering that CIS may be a precursor stage to malignant tumors the risk factors may be shared and for many environmental risk factors this appears to be the case^2^. Data on relative risks for breast, melanoma and some other CISs in families of concordant invasive cancer suggest that familial risk for CIS may be approximately equal to familial risks between the invasive forms of these cancers but data on concordant CISs are limited^6–8^. In the present study we assess familial risks of anogenital (cervical, other female and male genital and anal) cancers and skin cancers between CISs types and between CIS and invasive forms in order to test the hypothesis of shared familial risk factors to be extended to CIS, for which data are lacking in many cancer registries. Our previous study on familial CIS published in 2008 included far lower case numbers (e.g., only ¼ for male genital and 1/3 for female genital CIS); furthermore, it was limited to genital cancers and the present inclusion of upper aerodigestive tract and colorectal cancers, melanoma and non-Hodgkin lymphoma is completely novel^7^. According to the International Classification of Diseases version 7, ‘other female genital cancers’ include the vulva and the vagina; ‘other male genital cancers’ include the penis and the scrotum. These CISs and cancers share the SCC histology^9^. Anogenital cancers are associated with human papillomavirus (HPV) infection, particularly related to the alpha genus of mucosal HPV types 16 and 18^10,11^. In skin SCC the beta genus HPVs may be playing a role but the evidence has not been equivocal^12,13^. Another common denominator for these cancers is increasing risk in cases of immunodeficiency^14–16^. The risk is high in immunosuppressed organ transplant patients^13–15^. As immune deficiency may be an important contributor even to cancers unrelated of infection, inclusion of non-Hodgkin lymphoma in the present study is relevant, as it is one of the most responsive cancers to immunosuppression^14,16,17^.

We use the Swedish Family-Cancer Database which is the largest family dataset in the world with a globally unique collection of CIS cases. We analyzed risks for both concordant (same cancer sites) and discordant (different cancer sites) CISs and also including invasive cancers of the cervix, other female genitals and skin. In order to internally confirm the results we assess familial risks bi-directionally (in reverse order) as explained in Methods. For parent-offspring relationships the bidirectional analyses are essentially independent and, when agreeing, such data would provide support for true biological associations.

## Results

The RRs were calculated to the 0–83 year old offspring generation for with 303,630 CIS cases were reported to the cancer registry (Supplementary Table 1). The largest numbers were for cervical (177,285, median diagnostic age 32 years) and skin (36,516, 68 years) CISs. The number of CISs in the anus was 330, in other female genital it was 3326 and in other male genital it was 1000. Considering also the parental generation the total CIS numbers reached 464,484.

Risks for concordant sites are shown in Table 1 for familial CIS-CIS and CIS-invasive cancer associations. RRs were calculated for daughters whose first-degree family members were diagnosed with concordant CIS or invasive cancer. For cervical CIS, 20,188 familial patients were detected, resulting in RR of 1.77. The RR was 1.41 for invasive cervical cancer when a family member was diagnosed with cervical CIS; in reverse order the RR for cervical CIS was 1.54 when a family was diagnosed with invasive cervical cancer. Note that the differences between CIS-CIS and CIS-invasive cancer analyses were significant, i.e., 95% confidence interval (CIs) did not overlap. For other female genital cancer only CIS-CIS association of RR 2.74 was significant. For skin SCC the RR for CIS-CIS association was 2.29 which was significantly higher than both of the CIS-invasive associations (1.92 and 2.07). For skin cancer male and female risks did not differ.

**Table 1:** Risks for *in situ* and invasive cancer when family members were diagnosed with concordant *in situ* or invasive cancer.

Cancer site family member	RR for *in situ* cancer when family had the same *in situ* cancer			RR for invasive cancer when family had same *in situ* cancer			RR for *in situ* cancer when family had the same invasive cancer		
	Cases	RR	95%CI	Cases	RR	95%CI	Cases	RR	95%CI
Cervix	20188	**1**.**77**	**1**.**75–1**.**80**	1127	**1**.**41**	**1**.**33–1**.**50**	2763	**1**.**54**	**1**.**48–1**.**60**
Other female genital	15	**2**.**74**	**1**.**65–4**.**56**	4	1.19	0.45–3.18	9	1.06	0.55–2.05
Skin squamous cell	3207	**2**.**29**	**2**.**21–2**.**38**	1620	**1**.**92**	**1**.**83–2**.**02**	2097	**2**.**07**	**1**.**98–2**.**17**
Male	1612	**2**.**30**	**2**.**19–2**.**42**	951	**1**.**96**	**1**.**83–2**.**10**	1047	**2**.**08**	**1**.**95–2**.**21**
Female	1595	**2**.**28**	**2**.**17–2**.**40**	669	**1**.**87**	**1**.**73–2**.**03**	1050	**2**.**07**	**1**.**95–2**.**21**

RR = relative risk, 95%CI = 95% confidence interval, bold font indicates that the lower limit of 95%CI does not include 1.00.

Discordant associations of cervical CIS are shown in Table 2. Bi-directional associations for cervical CIS cancer were found for other female genital CIS (1.59 and 1.79) and non-Hodgkin lymphoma (1.24 and 1.36). Among CIS-invasive pairs none were bi-directionally significant but the risk for female genital CIS was increased (1.42) when a family member had invasive cervical cancer; for upper aerodigestive tract CIS the RR was 1.57 when a family member had invasive cervical cancer. The bottom line shows the RRs for all CISs or invasive cancers combined, with significant differences between respective CIS-CIS pairs and invasive-CIS pairs (1.35 vs. 1.12), and CIS-CIS pairs and CIS-invasive pairs (1.52 vs 1.31). When relevant, sex-specific risks were considered but none of the differences were significant (95%CIs overlapped).

**Table 2:** Risks for *in situ* and invasive cervical cancers in families of any *in situ* cancer, and risk for any *in situ* cancers in families of *in situ* and invasive cervical cancers.

Cancer site family member	RR for *in situ* cervical cancer when family member had *in situ* cancer			RR for *in situ* cancer when family member had *in situ* cervical cancer			RR for cervical cancer when family member had *in situ* cancer			RR for *in situ* cancer when family member had cervical cancer		
	Cases	RR	95%CI	Cases	RR	95%CI	Cases	RR	95%CI	Cases	RR	95%CI
Other female genital	458	**1**.**59**	**1**.**45–1**.**74**	347	**1**.**79**	**1**.**60–2**.**01**	26	1.22	0.83–1.80	51	**1**.**42**	**1**.**08–1**.**87**
Skin, squamous cell	5966	0.93	0.91–0.96	1645	1.03	0.98–1.08	427	0.86	0.78–0.94	378	0.99	0.90–1.10
Male	NR			806	1.04	0.97–1.12	NR			198	1.06	0.92–1.21
Female	As above			839	1.01	0.95–1.09	As above			180	0.93	0.81–1.08
Upper aerodigestive tract	243	1.08	0.96–1.23	86	**1**.**26**	**1**.**01–1**.**57**	22	1.24	0.82–1.89	24	**1**.**57**	**1**.**05–2**.**36**
Male	NR			51	1.15	0.87–1.53	NR			13	1.30	0.75–2.26
Female	As above			35	**1**.**44**	**1**.**02–2**.**04**	As above			11	**2**.**06**	**1**.**13–3**.**75**
Other male genital	117	**1**.**27**	**1**.**06–1**.**52**	56	1.00	0.76–1.31	9	1.33	0.69–2.55	16	1.57	0.96–2.57
Colorectum	2223	0.97	0.93–1.02	819	0.99	0.92–1.06	181	1.03	0.89–1.19	185	0.98	0.85–1.13
Male	NR			476	1.02	0.93–1.12	NR			113	1.05	0.87–1.26
Female	As above			343	0.95	0.85–1.06	As above			72	0.89	0.70–1.12
Melanoma	1850	0.98	0.94–1.03	1283	**1**.**09**	**1**.**03–1**.**15**	122	0.93	0.78–1.11	219	0.97	0.85–1.11
Male	NR			541	1.02	0.93–1.11	NR			112	1.04	0.86–1.25
Female	As above			742	**1**.**14**	**1**.**06–1**.**23**	As above			107	0.90	0.74–1.09
Non-Hodgkin lymphoma	144	**1**.**24**	**1**.**05–1**.**46**	65	**1**.**36**	**1**.**06–1**.**75**	12	1.34	0.76–2.36	11	1.09	0.60–1.97
Male	NR			40	**1**.**47**	**1**.**06–2**.**03**	NR			5	0.85	0.35–2.05
Female	As above			25	1.22	0.81–1.83	As above			6	1.42	0.64–3.19
All (including cervix)	33607	**1**.**35**	**1**.**33–1**.**37**	26966	**1**.**52**	**1**.**50–1**.**54**	2090	**1**.**12**	**1**.**07–1**.**18**	4089	**1**.**31**	**1**.**27–1**.**36**
Male	NR			2474	**1**.**05**	**1**.**01–1**.**09**	NR			558	1.06	0.97–1.15
Female	As above			24492	**1**.**58**	**1**.**56–1**.**60**	As above			3531	**1**.**36**	**1**.**31–1**.**40**

RR = relative risk, 95%CI = 95% confidence interval, bold font indicates that the lower limit of 95%CI does not include 1.00.

In Table 3, discordant familial risks for other female genital CIS-CIS analyses showed increased bi-directional associations for cervical (RRs 1.79 and 1.59), skin (1.25 and 1.38), other male genital (2.82 and 3.79) and ovarian CIS (1.66 and 1.85). Among CIS-invasive pairs the only bidirectional association was noted with cervical cancer (1.48 and 1.19 which was significantly lower than 1.59 for the comparable CIS-CIS analysis). In the bottom line significant differences between respective combined CIS-CIS pairs and invasive-CIS pairs are shown (1.48 vs. 1.15), similar to CIS-CIS pairs and CIS-invasive pairs (1.46 vs 1.13). Sex-specific risks were not significant.

**Table 3:** Risks for *in situ* and invasive other female genital cancers in families of any *in situ* cancer, and risk for any *in situ* cancers in families of *in situ* and invasive other female genital cancers.

Cancer site family member	RR for *in situ* female genital cancer when family member had *in situ* cancer			RR for *in situ* cancer when family member had *in situ* female genital cancer			RR for female genital cancer when family member had *in situ* cancer			RR for *in situ* cancer when family member had female genital cancer		
	Cases	RR	95%CI	Cases	RR	95%CI	Cases	RR	95%CI	Cases	RR	95%CI
Cervix	347	**1**.**79**	**1**.**60–2**.**01**	458	**1**.**59**	**1**.**45–1**.**74**	166	**1**.**48**	**1**.**27–1**.**74**	475	**1**.**19**	**1**.**09–1**.**30**
Skin, squamous cell	163	**1**.**25**	**1**.**06–1**.**46**	73	**1**.**38**	**1**.**09–1**.**73**	86	1.01	0.81–1.25	121	1.15	0.96–1.38
Male	NR			45	**1**.**69**	**1**.**26–2**.**26**	NR			52	1.00	0.76–1.31
Female	As above			28	1.06	0.73–1.53	As above			69	1.30	1.03–1.65
Upper aerodigestive tract	8	1.73	0.86–3.45	4	1.86	0.70–4.95	5	1.64	0.68–3.95	3	0.75	0.24–2.34
Male	NR			3	2.13	0.69–6.62	NR			3	1.16	0.37–3.60
Female	As above			1	1.33	0.19–9.46	As above			0	—	—
Other male genital	5	**2**.**82**	**1**.**17–6**.**78**	6	**3**.**79**	**1**.**70–8**.**46**	2	1.83	0.46–7.34	4	1.59	0.60–4.26
Colorectum	39	0.87	0.63–1.19	25	0.95	0.64–1.41	27	0.92	0.63–1.35	43	0.85	0.63–1.15
Male	NR			14	**1**.**69**	**1**.**26–2**.**26**	NR			26	0.90	0.61–1.32
Female	As above			11	0.98	0.55–1.78	As above			17	0.79	0.49–1.27
Ovary	16	**1**.**66**	**1**.**02–2**.**71**	17	**1**.**85**	**1**.**15–2**.**98**	5	0.81	0.34–1.95	18	1.20	0.75–1.90
Melanoma	31	0.90	0.63–1.28	37	1.09	0.79–1.50	11	0.55	0.30–1.00	55	0.96	0.74–1.25
Male	NR			15	0.93	0.56–1.54	NR			32	1.14	0.81–1.62
Female	As above			22	1.23	0.81–1.86	As above			23	0.79	0.52–1.19
Non-Hodgkin lymphoma	2	0.89	0.22–3.54	2	1.39	0.35–5.56	0	—	—	0	—	—
Male	NR			1	1.19	0.17–8.45	0	—	—	0	—	—
Female	As above			1	1.67	0.24–11.91	0	—	—	0	—	—
All (including female genital)	668	**1**.**48**	**1**.**36–1**.**62**	697	**1**.**46**	**1**.**35–1**.**57**	330	**1**.**15**	**1**.**03–1**.**30**	839	**1**.**13**	**1**.**05–1**.**21**
Male	NR			102	**1**.**34**	**1**.**11–1**.**63**	NR			153	1.08	0.92–1.27
Female	As above			595	**1**.**47**	**1**.**36–1**.**60**	As above			686	**1**.**14**	**1**.**05–1**.**22**

RR = relative risk, 95%CI = 95% confidence interval, bold font indicates that the lower limit of 95%CI does not include 1.00

Similar discordant analyses were carried out with CIS and invasive SCC skin cancer (Table 4). Bi-directional CIS-CIS associations were observed with skin CIS and other female genital (1.38 and 1.25), upper aerodigestive tract (1.48 and 1.33), skin melanoma (1.63 and 1.59) CISs. The two latter ones showed also bi-directional associations in CIS-invasive pairs which however were of the same magnitude as the respective CIS-CIS associations. In contrast to the previous tables there was no significant difference between the combined CIS-CIS and CIS-invasive cancer RRs in the bottom line.

**Table 4:** Risks for *in situ* and invasive skin squamous cell cancers in families of any *in situ* cancer, and risk for any *in situ* cancers in families of *in situ* and invasive skin squamous cell cancers.

Cancer site family member	RR for *in situ* skin squamous cell cancer when family member had *in situ* cancer			RR for *in situ* cancer when family member had *in situ* skin squamous cell cancer			RR for skin squamous cell cancer when family member had *in situ* cancer			RR for *in situ* cancer when family member had skin squamous cell cancer		
	Cases	RR	95%CI	Cases	RR	95%CI	Cases	RR	95%CI	Cases	RR	95%CI
Cervix	1645	1.03	0.98–1.08	5966	0.93	0.91–0.96	1106	**1**.**13**	**1**.**06–1**.**20**	4201	0.98	0.95–1.02
Male	806	1.04	0.97–1.12	NR			633	**1**.**15**	**1**.**06–1**.**24**	NR		
Female	839	1.01	0.95–1.09	As above			473	**1**.**10**	**1**.**00–1**.**21**	As above		
Other female genital	73	**1**.**38**	**1**.**09–1**.**73**	163	**1**.**25**	**1**.**06–1**.**46**	46	**1**.**42**	**1**.**06–1**.**90**	102	1.13	0.93–1.38
Male	45	**1**.**69**	**1**.**26–2**.**26**	NR			28	**1**.**51**	**1**.**04–2**.**19**	NR		
Female	28	1.06	0.73–1.53	As above			18	1.34	0.84–2.13	As above		
Upper aerodigestive tract	74	**1**.**48**	**1**.**18–1**.**86**	72	**1**.**33**	**1**.**05–1**.**69**	44	**1**.**49**	**1**.**11–2**.**00**	55	**1**.**44**	**1**.**10–1**.**89**
Male	35	**1**.**45**	**1**.**04–2**.**01**	46	1.31	0.98–1.77	26	**1**.**56**	**1**.**06–2**.**29**	24	0.96	0.64–1.45
Female	39	**1**.**52**	**1**.**11–2**.**08**	26	1.35	0.91–2.00	18	1.40	0.88–2.23	31	**2**.**33**	**1**.**62–3**.**35**
Other male genital	25	1.44	0.97–2.13	37	0.97	0.70–1.35	14	1.35	0.80–2.29	26	1.00	0.68–1.48
Male	16	**1**.**93**	**1**.**18–3**.**15**	As above			9	1.57	0.82–3.01	As above		
Female	9	0.99	0.52–1.90	NR			5	1.10	0.46–2.64	NR		
Colorectum	482	1.03	0.94–1.13	651	0.93	0.86–1.00	296	1.06	0.94–1.18	427	0.86	0.78–0.95
Male	227	0.98	0.86–1.11	352	0.87	0.78–0.97	172	1.07	0.92–1.25	249	0.88	0.77–0.99
Female	255	1.08	0.95–1.22	299	1.00	0.89–1.13	124	1.04	0.87–1.24	178	0.84	0.72–0.97
Melanoma	517	**1**.**63**	**1**.**49–1**.**78**	1371	**1**.**59**	**1**.**51–1**.**68**	296	**1**.**54**	**1**.**38–1**.**73**	896	**1**.**50**	**1**.**40–1**.**60**
Male	250	**1**.**59**	**1**.**40–1**.**80**	651	**1**.**56**	**1**.**44–1**.**69**	159	**1**.**45**	**1**.**24–1**.**70**	442	**1**.**53**	**1**.**39–1**.**68**
Female	267	**1**.**67**	**1**.**48–1**.**88**	720	**1**.**62**	**1**.**50–1**.**75**	137	**1**.**67**	**1**.**41–1**.**97**	454	**1**.**47**	**1**.**34–1**.**62**
Non-Hodgkin lymphoma	28	1.17	0.81–1.70	40	1.09	0.79–1.50	19	1.33	0.85–2.09	25	0.97	0.65–1.44
Male	12	1.03	0.59–1.82	26	1.20	0.81–1.79	11	1.36	0.75–2.45	13	0.85	0.49–1.48
Female	16	1.31	0.80–2.14	14	0.93	0.54–1.58	8	1.30	0.65–2.61	12	1.14	0.64–2.03
All (including SCC^a^)	6424	**1**.**50**	**1**.**46–1**.**55**	13348	**1**.**18**	**1**.**16–1**.**20**	3677	**1**.**41**	**1**.**36–1**.**46**	9009	**1**.**17**	**1**.**14–1**.**19**
Male	3180	**1**.**51**	**1**.**45–1**.**57**	3099	**1**.**56**	**1**.**51–1**.**62**	2121	**1**.**43**	**1**.**36–1**.**50**	2060	**1**.**47**	**1**.**41–1**.**54**
Female	3244	**1**.**50**	**1**.**44–1**.**56**	10249	**1**.**10**	**1**.**08–1**.**12**	1556	**1**.**39**	**1**.**32–1**.**47**	6949	**1**.**10**	**1**.**07–1**.**12**

SCC^a^ = skin squamous cell cancer.

RR = relative risk, 95%CI = 95% confidence interval, bold font indicates that the lower limit of 95%CI does not include 1.00.

We carried out detailed analyses for two rare CIS types, other male genital and anal CIS but because of low case numbers no data are shown. The only significant bi-directional association was found with other female genital CIS; the RR for other male genital CIS was 3.79 (N = 6; 95%CI 1.70–8.46) when a family member was diagnosed with other female genital CIS, and it was 2.82 (N = 5; 95%CI 1.17–6.78) for female genital CIS when a family member was diagnosed with male genital CIS. For anal CIS-CIS pairs no bi-directional associations were found. Invasive anal cancer was associated with cervical CIS (RR 1.39; N = 131, 95%CI 1.16–1.66) and cervical CIS was associated with invasive anal cancer (RR 1.44; N = 327, 95%CI 1.29–1.60). Individual associations were found for anal CIS with male and female genital cancers and with non-Hodgkin lymphoma.

## Discussion

In this systematic assessment of familial risks among CISs and between CIS and invasive anogenital and skin cancers several novel observations emerged. For three CISs we showed concordant bi-directional associations, including also associations between CIS and invasive cancer at the same sites. Additionally, we could define consistent clustering between CIS at discordant sites and some were even supported by associations with invasive cancers. It is likely that the clustering can be explained at least in part by shared HPV infections and/or deficiencies in host immune response to infections^7^.

The concordant risks for CIS were 2.74 for female genitals, 1.77 for cervical and 2.29 for SCC skin CISs. Risks for concordant association between CIS and invasive cancers were consistently lower than for concordant CIS-CIS associations. For other female genital cancer, CIS-invasive cancer associations were not significant but the case numbers were low. For cervical and skin sites, CIS-CIS associations were significantly higher than the respective CIS-invasive cancer associations (1.41 and 1.54, the bi-directional risk for cervical CIS-invasive; 1.92 and 2.07, the bi-directional risk for skin CIS-invasive). The low case numbers for other female genital cancers do not allow conclusions. On contrary, the large case numbers for cervical and skin CIS and invasive cancers suggest that the CIS and invasive types share familial risk factors to some degree but these appear not to be identical. These data appear consistent with the model that CIS may be a precursor of invasive cancer but the progressing clone of cells has acquired properties distinguishing them from the precursor population. For example, a clone of cells with specific HPV mutations will allow escape from host immune surveillance.

For comparison, in our recent analysis of invasive cancers, concordant other female genital cancers showed an RR of 2.72, almost exactly as the present CIC-CIS result of 2.74 (unpublished). According to the literature, for concordant invasive cervical cancer familial associations have been 1.80 (between sisters) and 1.74 (between daughter and mother)^7^. For invasive skin SCC concordant familial association has been reported as 2.06^18^.

Cervical CIS associated with female genital and anal CIS (and to some degree with invasive types) which would be consistent with susceptibility to HPV infections. Cervical CIS was also consistently associated with non-Hodgkin lymphoma. Based on cancers arising in immunosuppressed patients, non-Hodgkin lymphoma is considered one of the hallmarks of dysfunctional immune system^14,15^. Thus HPV susceptibility may be assisted by weak immune resistance shared by family members. HPV infections are usually sexually transmitted which would be unlikely to explain familial risk, and non-sexual transmissions from an infected mother during pregnancy or early childhood are supposed to be rare^19,20^. We assume that shared life-style with many sexual partners may the explanation to the observed familial risks. In our previous study on women who had children with different men in Sweden we observed increased risks for many life-style related cancers, including cervical, other female genital, upper aerodigestive tract, anal, liver, and lung cancers^21^. The results would thus suggest a gene-environment association in which immune dysfunction would be the genetic partner and HPV the environmental actor.

It is noteworthy that there was no association between cervical and skin CISs for which the infective HPV genus is suggested to be different and ultraviolet irradiation is the risk factor for skin SCC only^12^. The case numbers were large and the lacking of risk was consistent in Tables 2 and 4. Yet skin CIS was associated with female genital and upper aerodigestive tract CISs, both of which are known targets of mucosal HPV types^12^. The explanation remains unclear but the diagnostic age for skin CIS (68 years) was more than doubled for that of cervical CIS (32 years). A curious bi-directional association was noted between ovarian CIS and other female genital CIS. Previous data on the possible role of HPV in ovarian cancer is inconclusive, based on small positive and negative studies^12^.

In conclusion, the results showed that anogenital CISs showed concordant familial associations and discordant associations between each other, most likely related to a life-style with risk of HPV infections. For cervical CIS association with non-Hodgkin lymphoma CIS suggested contribution by immune dysfunction. Familial risks were lower for concordant CIS-invasive cancer associations than for CIS-CIS associations which suggests only a partial overlap of risk factors for the CIS and invasive cancers.

## Methods

### Database and cancer ascertainment

We used the update of the Swedish Family-Cancer Database which covered CIS and invasive cancers from 1958 through 2015 and family links over a century^22^. The Database includes 16 million individuals, covering the offspring generations born between 1932 and 2015 and their biological parents (the parental generation) in some 4 million nuclear families. Siblings could be identified only in the offspring generation which reached a maximal age of 83 years in 2015.

Coverage of cytologically or histologically verified incident cancers is considered to be over 90% complete on account of compulsory nationwide registration by clinicians and pathologists^23^. Cancer identification was based on the four-digit code according to the 7^th^ revisions of the International Classification of Diseases (ICD-7).

### Statistical analysis

Relative risks (RRs) were used to estimate cancer risks and each table contained three or four different but related analyses. RRs were calculated for the offspring generation. The first pair of data included CIS-CIS risks: (1) risk for CIS X when family members (parents of siblings) were diagnosed with any defined CIS, and (2) risk for any defined CIS and risk when family members were diagnosed with CIS X. For concordant CISs (Table 1) the results for (1) and (2) were identical and the data were not repeated. The second pair of data included invasive cancer-CIS: (3) risk for cancer X in offspring when family members were diagnosed with any defined CIS, and 4) risk of any defined CIS when family members were diagnosed with cancer X. Analyses (1) and (2) as well as (3) and (4) are independent for discordant cancers, and positive results in both analyses provided strong support for a true association. The differences between analyses (1) and (3), or (2) and (4) differ in that invasive cancer at the same site is replaced by CIS, and is being used to compare RRs between CIS-CIS and CIS-invasive associations.

Follow-up was started for each offspring at birth, immigration or January 1^st^, 1958, whichever came latest. Follow-up was terminated on diagnosis of CIS or invasive cancer, death, emigration, or the closing date of the study, which was December 31^st^, 2015. Termination of follow-up at diagnosis of the first tumor is a standard practice in cancer epidemiology to guard against biases such as surveillance bias^24^. The reference rates were generated for the same CIS or invasive cancer for those who did not have the relevant family history. Poisson regression modeling was employed to estimate RRs and corresponding 95% confidence intervals (CI). Potential confounders, including sex, age group (5-year bands), period (5-year bands), socioeconomic status (blue-collar worker, white-collar worker, farmer, private, professional, or other/unspecified) and residential area (large cities, South Sweden, North Sweden, or unspecified) were added to the model as covariates.

All statistical analyses were done with the SAS version 9.4. Data are shown in tables for anogenital (cervix, other female and male genital, upper aerodigestive tract) and skin CISs, for common CIS types (breast and colorectum) and for any bi-directional associations (ovary in Table 3).

### Ethical statement

The study was approved by the Ethical Committee of Lund University (Reg.nr 2012/795), Sweden, and the study was conducted in accordance with the approved guidelines not requesting informed consent. The study is national register-based study on anonymous personal data.

## Supplementary information

Dataset1

## References

[1] Kumar, V., Cotran, R. & Robbins, S. *Basic Pathology*, 1–775 (W. B. Saunders, Philadelphia, 1997).

[2] Voltaggio L (2016). Current concepts in the diagnosis and pathobiology of intraepithelial neoplasia: A review by organ system. CA Cancer J Clin.

[3] Hussain SK, Sundquist J, Hemminki K (2010). Incidence trends of squamous cell and rare skin cancers in the Swedish national cancer registry point to calendar year and age-dependent increases. J Invest Dermatol.

[4] Centre for Epidemiology. *Cancer incidence in Sweden 2012*, (The National Board of Health and Welfare, Stockholm, 2013).

[5] Pukkala E (2018). Nordic Cancer Registries - an overview of their procedures and data comparability. Acta Oncol.

[6] Chen T (2014). Multiple primary (even *in situ*) melanomas in a patient pose significant risk to family members. Eur J Cancer.

[7] Hussain SK, Sundquist J, Hemminki K (2008). Familial clustering of cancer at human papillomavirus-associated sites according to the Swedish Family-Cancer Database. Int J Cancer.

[8] Lorenzo Bermejo J, Hemminki K (2005). Familial risk of cancer shortly after diagnosis of the first familial tumor. J Natl Cancer Inst.

[9] Tavassoli, F. & Devilee, P. (eds). *Tumours of the breast and female genital organs*, 432 (IARC Press, Lyon, 2003).

[10] Zur Hausen H (2009). The search for infectious causes of human cancers: where and why. Virology.

[11] Oh JK, Weiderpass E (2014). Infection and cancer: global distribution and burden of diseases. Ann Glob Health.

[12] IARC. Biological agents (2012). Volume 100 B. A review of human carcinogens. IARC Monogr Eval Carcinog Risks Hum.

[13] Bouwes Bavinck JN (2018). Human papillomavirus and posttransplantation cutaneous squamous cell carcinoma: A multicenter, prospective cohort study. Am J Transplant.

[14] Birkeland S (1995). Cancer risk after renal transplantation in the Nordic countries, 1964-1986. Int J Cancer.

[15] Wimmer CD (2007). The janus face of immunosuppression - de novo malignancy after renal transplantation: the experience of the Transplantation Center Munich. Kidney Int.

[16] Rama I, Grinyo JM (2010). Malignancy after renal transplantation: the role of immunosuppression. Nat Rev Nephrol.

[17] Hortlund M (2017). Cancer risks after solid organ transplantation and after long-term dialysis. Int J Cancer.

[18] Hussain SK, Sundquist J, Hemminki K (2009). The effect of having an affected parent or sibling on invasive and *in situ* skin cancer risk in Sweden. J Invest Dermatol.

[19] Syrjanen S (2010). Current concepts on human papillomavirus infections in children. Apmis.

[20] Koskimaa HM (2012). Human papillomavirus genotypes present in the oral mucosa of newborns and their concordance with maternal cervical human papillomavirus genotypes. J Pediatr.

[21] Li X, Hemminki K (2002). Cancer risks in women who had children with different partners from the Swedish Family-Cancer Database. Eur J Cancer Prev.

[22] Hemminki K, Ji J, Brandt A, Mousavi SM, Sundquist J (2010). The Swedish Family-Cancer Database 2009: Prospects for histology-specific and immigrant studies. Int J Cancer.

[23] Ji J, Sundquist K, Sundquist J, Hemminki K (2012). Comparability of cancer identification among Death Registry, Cancer Registry and Hospital Discharge Registry. Int J Cancer.

[24] Hemminki K (2017). Surveillance bias in cancer risk after unrelated medical conditions: example urolithiasis. Sci Rep.

