# Supplementary material for: Comparison of Familial Clustering of Anogenital and Skin Cancers Between In Situ and Invasive Types
Source: Sci Rep. 2019 Nov 6;9:16151. doi: 10.1038/s41598-019-51651-6 (PMC6834624; doi:10.1038/s41598-019-51651-6)
Supplement: Supplementary file 1 — Dataset1 [file 41598_2019_51651_MOESM1_ESM.docx]

COMPARISON OF FAMILIAL CLUSTERING OF ANOGENITAL AND SKIN CANCERS BETWEEN IN SITU AND INVASIVE TYPES

Luyao Zhang ^1,2^, Otto Hemminki ^3,4^, Guoqiao Zheng ^1,2^, Asta Försti ^1,5^, Kristina Sundquist ^5,6,7^ Jan Sundquist ^5,6,7^ and Kari Hemminki ^1,5^

**Supplementary Table 1** The number and age at diagnosis of patients with all in situ cancers

| **In situ cancer sites** | **Number of cancers in offspring** | **Median age at diagnosis in offspring (years)** | **Number of cancers in all** | **Median age at diagnosis in all (years)** |
| --- | --- | --- | --- | --- |
| Upper aerodigestive tract | 1390 | 61 | 3819 | 68 |
| Salivary glands | 113 | 44 | 501 | 55 |
| Esophagus | 380 | 64 | 837 | 72 |
| Stomach | 646 | 64 | 2327 | 75 |
| Small intestine | 357 | 64 | 624 | 71 |
| Colorectum | 17350 | 65 | 36442 | 72 |
| Anus | 330 | 53 | 549 | 63 |
| Liver | 524 | 65 | 1587 | 76 |
| Pancreas | 399 | 65 | 1346 | 75 |
| Nose | 860 | 52 | 1282 | 59 |
| Lung | 614 | 57 | 2952 | 72 |
| Breast (female and male) | 18468 | 55 | 25603 | 60 |
| Cervix | 177285 | 32 | 199811 | 33 |
| Endometrium | 1047 | 55 | 2518 | 59 |
| Uterus, unspecified | 5826 | 28 | 6194 | 29 |
| Ovary | 5718 | 50 | 8752 | 56 |
| Other female genital | 3326 | 49 | 4903 | 56 |
| Prostate | 3793 | 65 | 6975 | 69 |
| Testis | 306 | 31 | 370 | 34 |
| Other male genital | 1000 | 53 | 1608 | 63 |
| Kidney | 76 | 63 | 377 | 78 |
| Melanoma | 21558 | 58 | 29182 | 64 |
| Skin, squamous cell | 36516 | 68 | 112212 | 77 |
| Eye | 125 | 46 | 251 | 66 |
| Nervous system | 15 | 33 | 21 | 45 |
| Thyroid gland | 371 | 47 | 726 | 57 |
| Bone | 443 | 29 | 559 | 34 |
| Connective tissue | 359 | 44 | 520 | 53 |
| Non-Hodgkin lymphoma | 973 | 63 | 1780 | 71 |
| Hodgkin lymphoma | 27 | 36 | 51 | 62 |
| Myeloma | 104 | 62 | 445 | 74 |
| Leukemia | 2731 | 62 | 7384 | 74 |
| CUP^1^ | 390 | 61 | 2036 | 73 |
| All | 303630 |  | 464484 |  |

CUP^1^=cancer of unknown primary
